# A communication hub for phosphoregulation of kinetochore-microtubule attachment

**DOI:** 10.1016/j.cub.2024.04.067

**Published:** 2024-05-21

**Authors:** Jacob A. Zahm, Stephen C. Harrison

**Affiliations:** 1. Department of Biological Chemistry and Molecular Pharmacology, Harvard Medical School, Boston, MA, 02115 USA; 2. Howard Hughes Medical Institute, Boston, MA 02115, USA

## Abstract

The Mps1 and Aurora B kinases regulate and monitor kinetochore attachment to spindle microtubules during cell division, ultimately ensuring accurate chromosome segregation. In yeast, the critical spindle attachment components are the Ndc80 and Dam1 complexes (Ndc80c and DASH/Dam1c, respectively). Ndc80c is a 600-Å long heterotetramer that binds microtubules through a globular “head” at one end and centromere-proximal kinetochore components through a globular knob at the other end. Dam1c is a heterodecamer that forms a ring of 16–17 protomers around the shaft of the single kinetochore microtubule in point-centromere yeast. The ring coordinates the approximately eight Ndc80c rods per kinetochore. In published work, we showed that a site on the globular “head” of Ndc80c, including residues from both Ndc80 and Nuf2, binds a bipartite segment in the long, C-terminal extension of Dam1. Results reported here show, both by in vitro binding experiments and by crystal structure determination, that the same site binds a conserved segment in the long N-terminal extension of Mps1. It also binds, less tightly, a conserved segment in the N-terminal extension of Ipl1 (yeast Aurora B). Together with results from experiments in yeast cells and from biochemical assays reported in two accompanying papers, the structures and graded affinities identify a communication hub for ensuring uniform bipolar attachment and for signaling anaphase onset.

## eTOC blurb

Zahm and Harrison combine binding studies and structure determinations to identify a common interaction site on the Ndc80 complex of yeast kinetochores for kinases Mps1 and Ipl1 and for the DASH subunit, Dam1. Sequences and alphafold predictions suggest a widely conserved regulatory hub on Ndc80c, consistent with data in accompanying articles.

## INTRODUCTION

Faithful chromosome segregation in cell division requires bipolar spindle attachment of all sister chromatid pairs. Tension between two sister kinetochores signals biorientation, as the chromosome arms are still cohesin-linked^1–3^. Multiple phosphorylation and dephosphorylation events, in which the protein kinases Mps1 and Aurora B (Ipl1 in *S. cerevisiae*) are instrumental^4–18^, transduce the signals reporting attachment/detachment or tension/no-tension, deferring anaphase onset until all kinetochore pairs have attached to opposite spindle poles (the “spindle assembly checkpoint”: SAC, reviewed in^19^). These kinases have interrelated and apparently redundant roles in the error correction needed to achieve uniform biorientation; that is, they participate actively in generating correct attachments, by modifying various kinetochore components, rather than simply reporting attached and unattached states^1,9,17,20,21^. Their activities are also important at stages of cell division other than the assembly of an anaphase-competent mitotic apparatus, adding further complexity to analysis of their specific functions.

The components of the spindle microtubule attachment apparatus in yeast are the Ndc80 and Dam1 complexes (Ndc80c and DASH/Dam1c, respectively). Ndc80c, a heterotetramer of Ndc80, Nuf2, Spc24, and Spc25, is a 600 Å long rod, largely two-chain coiled-coil, with an Ndc80/Nuf2 globular “head” at one end and an Spc24/Spc25 globular knob at the other^22,23^. The Ndc80 and Nuf2 globular regions are both calponin-homology (CH) domains^24,25^. Together with an approximately 100-residue, N-terminal extension, the Ndc80 CH domain forms the principal microtubule contact^26,27^. DASH/Dam1c is a heterodecamer, about 17 copies of which form a ring around the microtubule shaft at the kinetochore (plus) end^28–31^. Through contacts from C-terminal extensions of four Dam1c components, Dam1, Ask1, and Spc19/Spc34, the ring organizes the multiple Ndc80 rods (estimated to be about eight:^32^) surrounding the single kinetochore microtubule^31,33^.

Major targets of Mps1 are a set of so-called MELT repeats on Spc105/Knl1 -- part of an intermediate, “adaptor” complex that links Ndc80c with centromere-proximal assemblies -- which in turn recruits the SAC signaling components^34,35^. Loss of Mps1 activity leads to prompt dephosphorylation of Spc105/Knl1 and therefore loss of a contribution to the SAC from the kinetochore in question^4,36^. Mps1 associates with the globular, Ndc80:Nuf2 head of Ndc80c, the main microtubule (MT) attachment component of kinetochores in nearly all eukaryotes^37,38^. Studies of the metazoan kinase have suggested that MTs and Mps1 compete for Ndc80c binding, leading to the view that binding of Mps1 reports an unattached Ndc80c and that attachment ejects it^37,38^. Experiments with isolated yeast kinetochores indicate that autophosphorylation of Mps1 may be sufficient to release it from Ndc80c^39^, and an accompanying paper^40^ shows directly that yeast Mps1 occupancy is compatible with microtubule binding, while also suggesting that autophosphorylation alone may not fully explain the dynamics of Mps1 in cells. The difference between the two sets of observations could reflect a difference between metazoan and yeast Mps1, as additional contacts with Ndc80 may be present in the former^38^, made by an Mps1 N-terminal segment that has no homologous representation in the latter^4^.

Substrates of Ipl1 in yeast include both Ndc80c (in particular, the N-terminal extension of Ndc80) and DASH/Dam1c^8,10,17,41,42^. Phosphorylation of certain Ser/Thr residues in these complexes weakens kinetochore attachments to microtubules. Absence of tension presumably favors Ipl1 access to these sites, thereby promoting error correction. In the case of syntely, for example, in which both sister chromatids are attached to the same pole, phosphorylation would weaken the contact, ultimately freeing one or both of the sister kinetochores to “try again” and reconnect correctly.

Ipl1/AuroraB is the catalytic component of the heterotetrameric chromosome passenger complex (CPC)^43^. A long, potentially extended connector, Sli15/INCENP, links Ipl1 at one end with two regulatory subunits, Bir1/Survivin and Nbl1/Borealin, at the other end. Bir1 dependent association of the CPC with centromeres during the onset of mitosis has led to the suggestion that the length of the INCENP “tether” could determine the likelihood of Ndc80 phosphorylation and that an increase under tension of the distance between centromeric chromatin and the Ipl1 sites on the Ndc80 N-terminal extension could be part of the tension-sensing mechanism^44^. Some studies in yeast have questioned this picture of a distance gradient, however, and an alternative model postulates local, tension-induced changes at the Ndc80-MT interface that sequester the Ipl1 target sites or reduce the frequency of their dissociation^45,46^.

Sorting out the interrelated functions of Mps1 and Ipl1 clearly requires direct structural pictures of their association with kinetochore components. We show here, biochemically and structurally, that short peptide segments on both Mps1 and Ipl1 bind the site on the Ndc80/Nuf2 head of Ndc80c that we have shown previously to bind an Ipl1-phosphoregulated peptide segment near the C-terminus of Dam1^47^. The segment of Mps1 that associates with Ndc80c is in the middle of the ~430-residue N-terminal extension of the kinase domain; the associating segment of Ipl1 is in a much shorter N-terminal extension of the kinase domain. The binding motifs are conserved in their key features in other point-centromere yeast.

In related work, Pleuger et al^40^ and Parnell et al^48^ have characterized Mps1:Ndc80c interactions both in vivo and in vitro; their analyses of mutant phenotypes together show the functional relevance of contacts in the Mps1:Ndc80c structure reported here. The common binding surface for Dam1, Mps1, and Ipl1 on the Ndc80c head is thus a central communication point for establishing the SAC, for triggering error correction and ultimately silencing the SAC, and for generating robust, bipolar attachments as the outcome.

## RESULTS

### Mps1 interaction with Ndc80c

**Figure 1.**
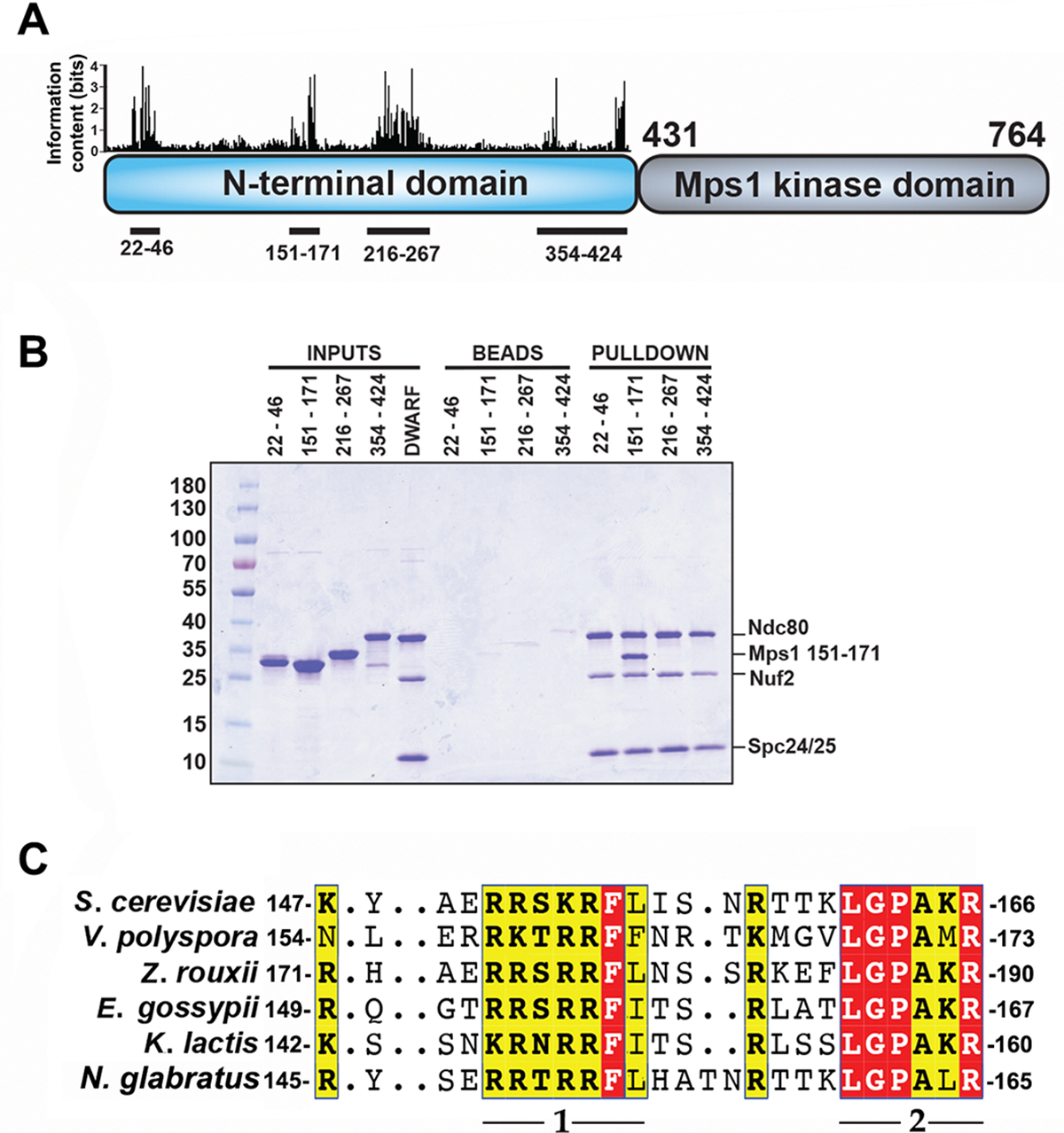
Binding of Mps1 N-terminal extension, residues 151–171, with Ndc80c^dwarf^. **(A)** Schematic representation of *S. cerevisiae* Mps1 domain structure. The bar graph above the N-terminal domain represents conservation determined by iterative searches with Jackhmmer^50^ and shown as “information content”, defined in ref^68^. Bar heights represent information content in bits, i.e., degree of conservation at a given position. These conserved stretches of sequence span the residues shown below the schematic. **(B)** Pulldown experiment testing which of the four conserved stretches in **(A)** bind Ndc80c^dwarf^. Ndc80c^dwarf^ was immobilized to saturation on Ni-NTA agarose and incubated with GST-fusions of each of the four conserved stretches. Each lane corresponds to the GST-fusion of the segment shown by the sequence interval in the label above the lane. **(C)** Multiple sequence alignment showing conservation of the binding motif among point-centromere yeast. Parts 1 and 2 of the bipartite binding motif are labeled below the alignment. Part 1 has both a basic region and a hydrophobic region; part 2 terminates in an invariant arginine. Red and yellow boxes show residues within this set that are fully conserved and conserved in character (basic, hydrophobic, etc.), respectively.

**Figure 2.**
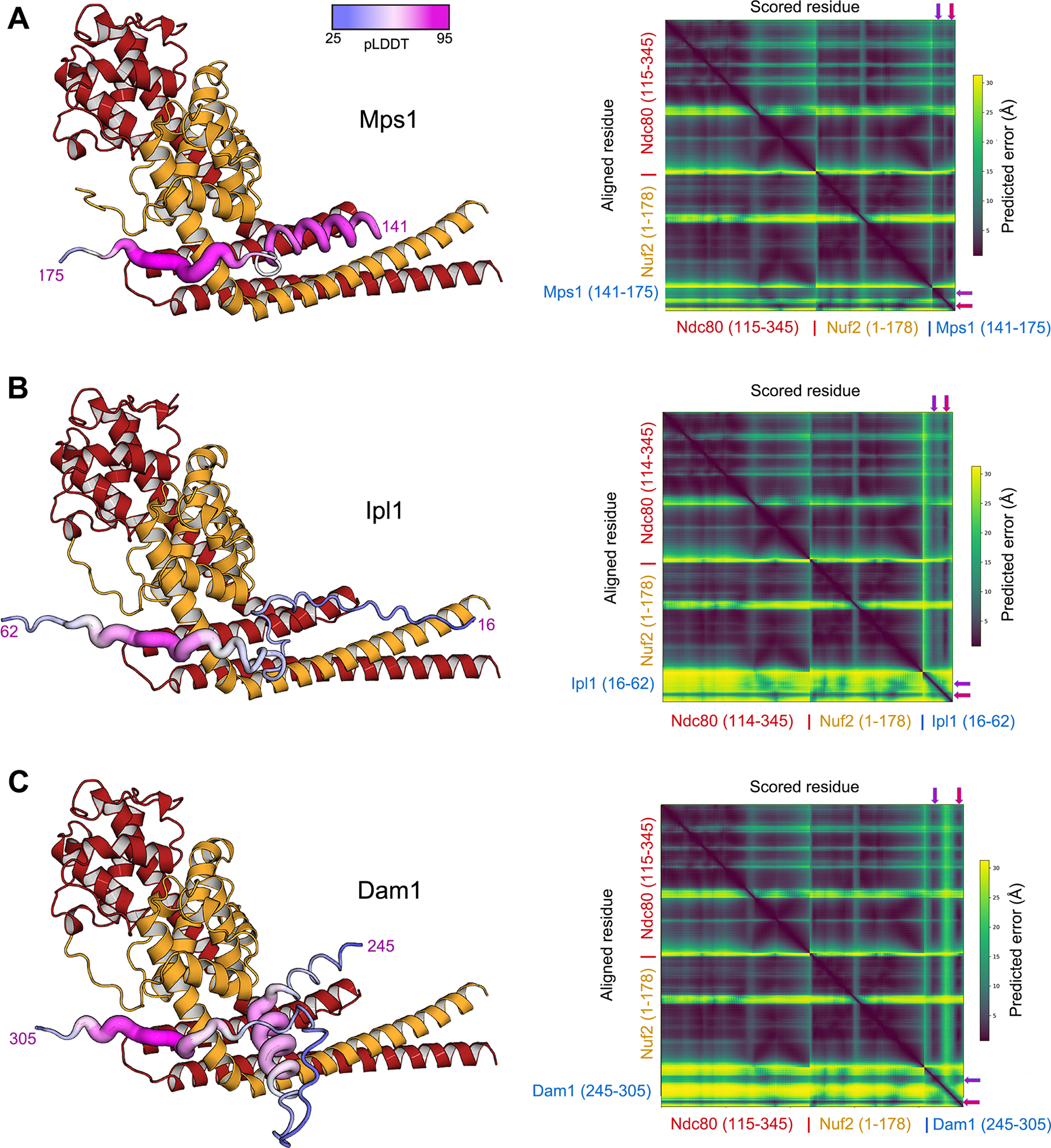
AF2 predictions and corresponding predicted alignment error (pAE) plots. Ndc80 and Nuf2 are in red and orange, respectively. The bound peptides are in colors indicated by the pLDDT local confidence metric scale at the top of panel (**A**). Magenta numerals indicate the first and last residues of the peptide segment, for (**A**) Mps1, (**B**) Ipl1, and (**C**) Dam1. The axes of the pAE plots are labeled with the molecular component and initial and final residues for that component. Red arrows indicate column and row corresponding to the invariant arginine; magenta arrows, to the the basic and hydrophobic residues in the first part of the bipartite motif. Rows indicate the residue on which the structure was aligned, and columns indicate the residue scored according to the scale on the right of each plot. For all three of the predictions, the predicted error for the invariant arginine and for residues just preceding is less than 5 Å for alignment on most of Ndc80 and Nuf2 (i.e. for columns close to those indicated by the red arrows). See also Figures S2, S3 and S5.

The long, N-terminal extension (residues 1–430) of yeast Mps1 has no immediately evident homology with sequences in metazoan Mps1 shown to interact with Ndc80c. Genetic analysis has suggested that residues 151–200 function in kinetochore biorientation, while residues 201–300 function in spindle-pole body duplication^49^. We examined conservation among the yeast orthologs by iterative searches with the program Jackhmmer^50^. Using the N-terminal 430 residues of *S. cerevisiae* Mps1 as query, we identified five segments conserved among point-centromere yeasts (Figure 1A). We carried out a set of pulldown experiments, expressing them as GST-fusions and incubating them with the “dwarf” Ndc80c construct (Ndc80c^dwarf^)^51^ immobilized on Ni-NTA agarose (Figure 1B). In this format, we expected avidity from GST dimerization and multivalent display of Ndc80c to enhance the assay sensitivity. Ndc80c captured only the GST fusion of a peptide containing residues 151–171 (Figure 1B, lane 12), which include the most conserved region with a consensus sequence (151)RRxRRF(I/L/F)(3–4x)RxxxLGPAxR(170)(Figure 1C). Lysine appears occasionally in place of arginine, except for the invariant R at the end of the motif. The consensus is an essentially bipartite motif, with a set of basic residues and two hydrophobic residues at one end connected by a segment of variable length to GPAxR at the other. A multi-chain Alphafold2 (AF2) prediction^52^ in which the input was Mps1 residues 150–200, Ndc80 residues 115–335, and Nuf2 residues 1–173 showed an interaction that included the consensus motif (Figure 2A). We used this prediction to restrict the length of the peptide used for binding experiments, as described in the next section, and to design the construct that yielded a crystal structure.

### Binding of Mps1 and Mps1 mutant peptides to Ndc80c

**Figure 3.**
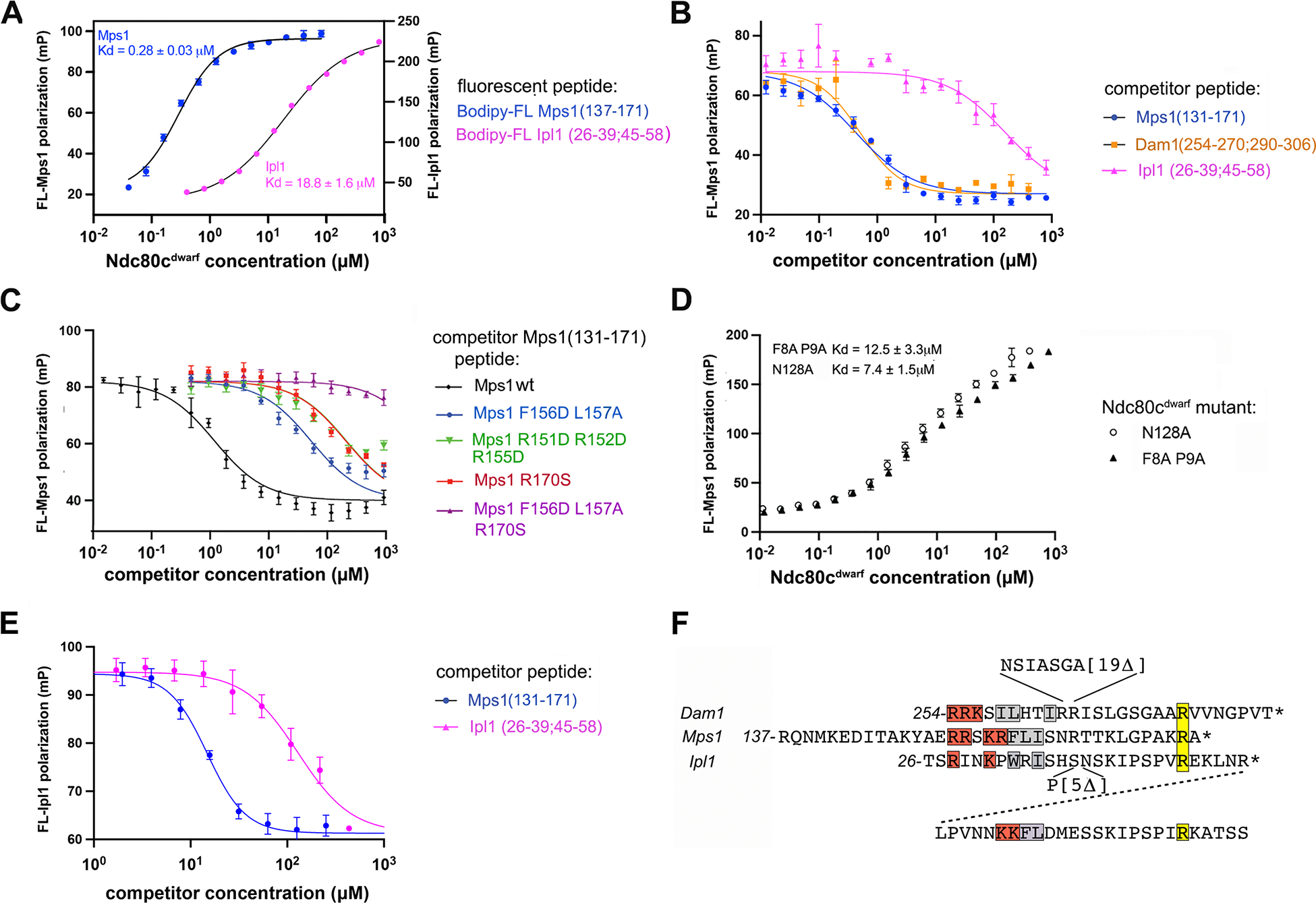
Fluorescence polarization measurements of Ndc80c^dwarf^ binding peptides from the N-terminal extensions of Mps1 and Ipl1. **(A)** Polarization of fluorescence from 20 nM Bodipy-FL Mps1(137–171) (Mps1 residues 137–171 plus a C-terminal cysteine modified with Bodipy-FL -- see Methods) and Bodipy-FL Ipl1 (26–39;45–59) as a function of Ndc80c^dwarf^ concentration. **(B)** Competition binding experiment: unlabeled competitor peptides (see figure annotation) were added in twofold serial dilutions to Bodipy-FL Mps1(137–171) and Ndc80c^dwarf^ at final concentrations of 20 nM and 500 nM respectively. Loss of polarization indicates displacement of bound Bodipy-FL Mps1(137–171) by the competitor. **(C)** As in **(B),** with competitor Mps1(137–171) peptides containing mutations that reduce affinity for Ndc80c^40^. **(D)** Polarization of fluorescence from 20 nM Bodipy-FL Mps1(137–171) as a function of the concentration of Ndc80c^dwarf^ bearing mutations in Nuf2 that interfere with its Mps1 interaction in cells^48^. (**E**) As in (**B**), but with 20 nM Bodipy-FL Ipl1 (26–39;45–59) as the fluorescent reporter and 27μM Ndc80c^dwarf^. (**F**) Alignment of Dam1, Mps1, and Ipl1 peptides used for experiments reported in (**A**)-(**E**). Basic and hydrophobic residues in the first part of the bipartite motif are in red and gray boxes, respectively; the invariant arginine in the second part is in a yellow box. For the Ipl1 peptide, the second copy of the motif, not used for the fluorescence polarization experiments, is shown in the bottom line.

We measured by fluorescence polarization the affinity of Ndc80^dwarf^, a shortened version of Ndc80c^51^, for a peptide comprising Mps1 residues 137–171, plus a C-terminal cysteine for labeling. We labeled the peptide with the Bodipy fluorophore and determined fluorescence anisotropy as a function of Ndc80c^dwarf^ concentration (Figure 3A). We then used unlabeled wild-type and mutant peptides in a competition format, at a fixed Ndc80c^dwarf^ concentration (Figures 3B,C). The mutant peptides corresponded to mutations characterized in one of the accompanying papers^40^. The wild-type Mps1 peptide displaced the fluorescent Mps1 peptide, at a concentration consistent with the affinity measured by direct titration. Mutating either the basic residues or the hydrophobic residues in the first part of the consensus motif or the conserved arginine at the end of the second part lowered affinity (raised the Ki) by roughly two logs; mutating both the final arginine and the hydrophobic residues eliminated any detectable competition.

We also determined the binding of the Mps1 peptide with two Ndc80c^dwarf^ constructs bearing mutations characterized in the other accompanying paper -- N128A and F8A P9A^48^. In both cases, the Mps1 peptide affinity was about 2 logs weaker than for the wild-type protein (Figure 3D).

### Structure of Ndc80c chimera with Mps1 binding motif

We validated conclusions from the competition binding experiments and AF2 prediction by determining an x-ray crystal structure of Ndc80c^dwarf^ associated with the corresponding peptide segments from Mps1. We applied the strategy we used to determine the Dam1-Ndc80c interaction^47^, by generating chimeras with the C-terminus of the Mps1 peptide joined to the N-terminus of Nuf2 as a continuous polypeptide chain. Because Ndc80c^dwarf^, designed originally to show the structure of the four-chain junction between the Ndc80:Nuf2 and Spc24:Spc25 heterodimeric components, crystallizes readily^51^, it is a useful scaffold for constructing these chimeric proteins.

**Figure 4.**
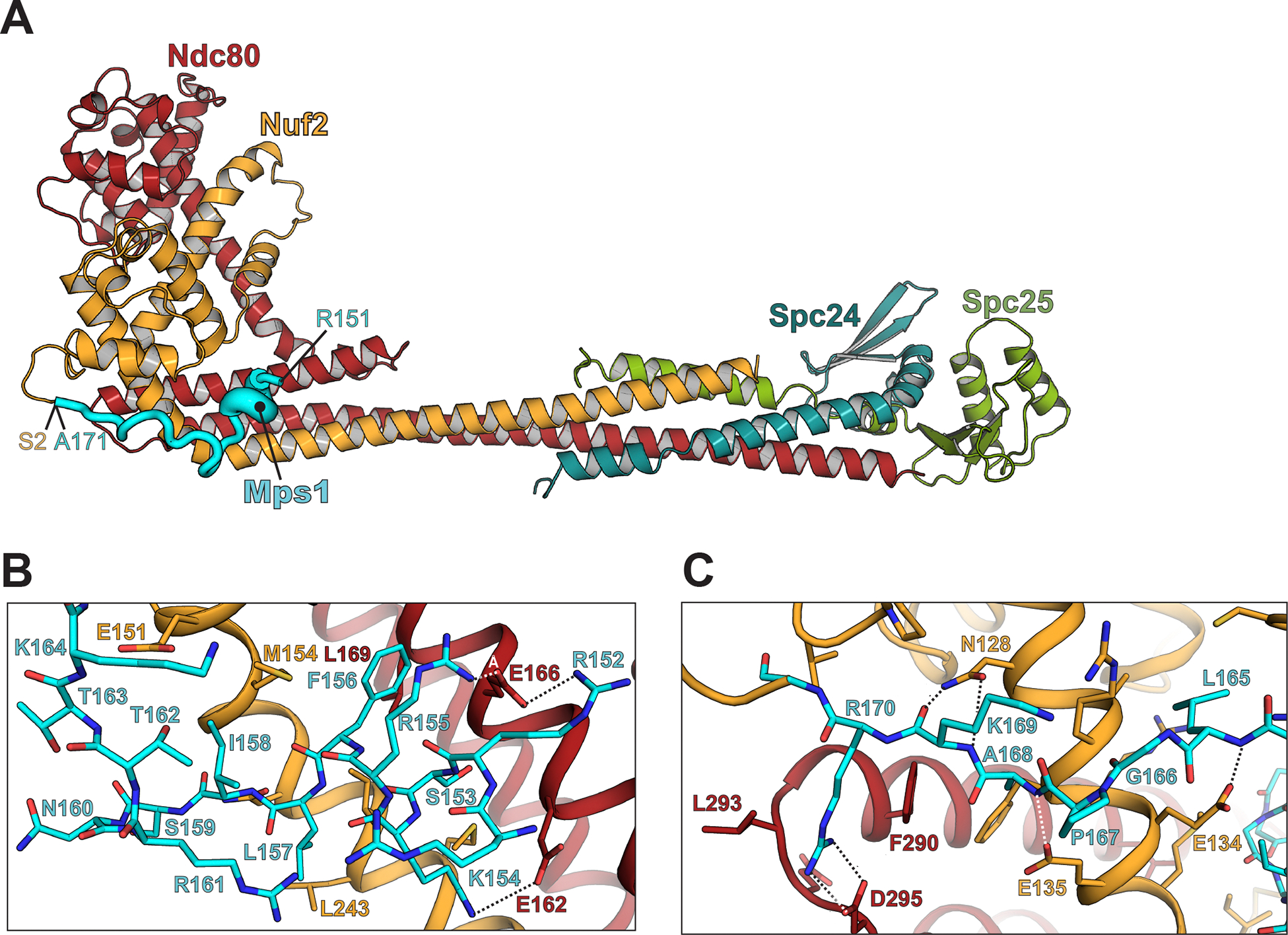
Crystal structure of Ndc80c^dwarf^ with Mps1 residues 124–176 fused to the Nuf2 N-terminus. **(A)** Cartoon representation of the Ndc80c^dwarf^ heterotetramer with the Mps1-Nuf2 fusion. Residue numbers show the positions of the initial Mps1-peptide residue (R151) that we could build with confidence and of the junction between the Mps1 segment and Nuf2 (A171/S2). **(B)** Close-up view of the helical N-terminal portion of the Mps1 segment (rotated about 60° clockwise from the view in **A**). (**C)** Close-up view of the C-terminal portion of the Mps1 segment (oriented approximately as in **A**). See also Figures S1, S2, S4, S5 and Table S1.

The Mps1: Ndc80c^dwarf^ chimera crystallized in space group C2221 (a = 72.5 Å, b = 168.5 Å, c = 229.6 Å). We recorded diffraction data to a minimum Bragg spacing of 2.9 Å and determined the structure as described in Methods (Figure 4A; Figure S1A; Table S1). Contacts in the structure are consistent with the conserved bipartite motif (Figure 1C) and with the conservation of residues in Ndc80:Nuf2 with which the two parts of the motif interact. Figure S2A shows a comparison of the structure with the AF2 prediction.

The N-terminal seven residues of the motif (RRSKRFL) form a short helix, which has a set of contacts with conserved glutamate residues in αH of Ndc80 and with conserved features of Nuf2 (Figure 3B). In the crystal structure, the guanidinium groups of R152 and R154 both bridge to the carboxylate of Ndc80 E166, and the side-chain NH3+ of K153 projects to the carboxylate of Ndc80 E162. The conserved phenylalanine, Mps1 F156, inserts into a hydrophobic pocket lined by Ndc80 L169, L170 and F172, and Nuf2 M154, with a further contribution from Mps1 L157 (Figure 4B).

The C-terminal six residues of the Mps1 motif (LGPAKR) extend across Nuf2 αG, with contacts from residues 128 and 130–132 (conserved in point-centromere yeast) (Figure 4C). N128, conserved not only in yeasts but also in metazoans, anchors the end of the motif with hydrogen bonds between its side-chain amide and the main-chain amide and carbonyl of K169 (the penultimate residue in the motif), thus mimicking the hydrogen bonds in an antiparallel β-sheet. This interaction positions the invariant arginine of the motif to salt bridge with Ndc80 D295, likewise conserved in both metazoans and yeast.

The structure rationalizes the phenotypes of mutants studied in the two accompanying papers, several of which we have incorporated into the binding measurements reported above. Our description of contacts with the N-terminal part of the bipartite motif can account for the effects of Mps1 mutations of basic and hydrophobic residues described in Pleuger et al^40^ (designated there as br and hm mutants, respectively). They have also examined phosphomimetic and alanine mutations at S159, T162, and T163, all of which are at the Mps1:Nuf2 interface. We expect that phosphorylation of T162 in particular, which faces a hydrophobic surface on Nuf2, at the junction between helices G and H, would displace that part of the bound peptide and presumably reduce affinity, but a displaced conformation would probably still allow the key residues in the binding motif to dock correctly.

Particularly strong phenotypes among the Nuf2 mutations studies by Parnell et al^48^ are at conserved residues F8 and P9 (mutated together), S124, and N128. We have described above the role of N128 in buttressing the bound peptide; it has the same key role in binding Dam1^47^. Residues F8 and P9 together bury the hydrogen-bonding interactions of N128 with the main chain of the penultimate residue in the Mps1 and Dam1 binding motifs and probably contribute both to the strength of those interactions and to correct positioning of the invariant arginine to salt bridge with Ndc80 D295. Phosphorylation of Nuf2 Ser124 would displace F8 and generally reposition the Nuf2 N-terminal loop.

### Ipl1 interaction with Ndc80c

**Figure 5.**
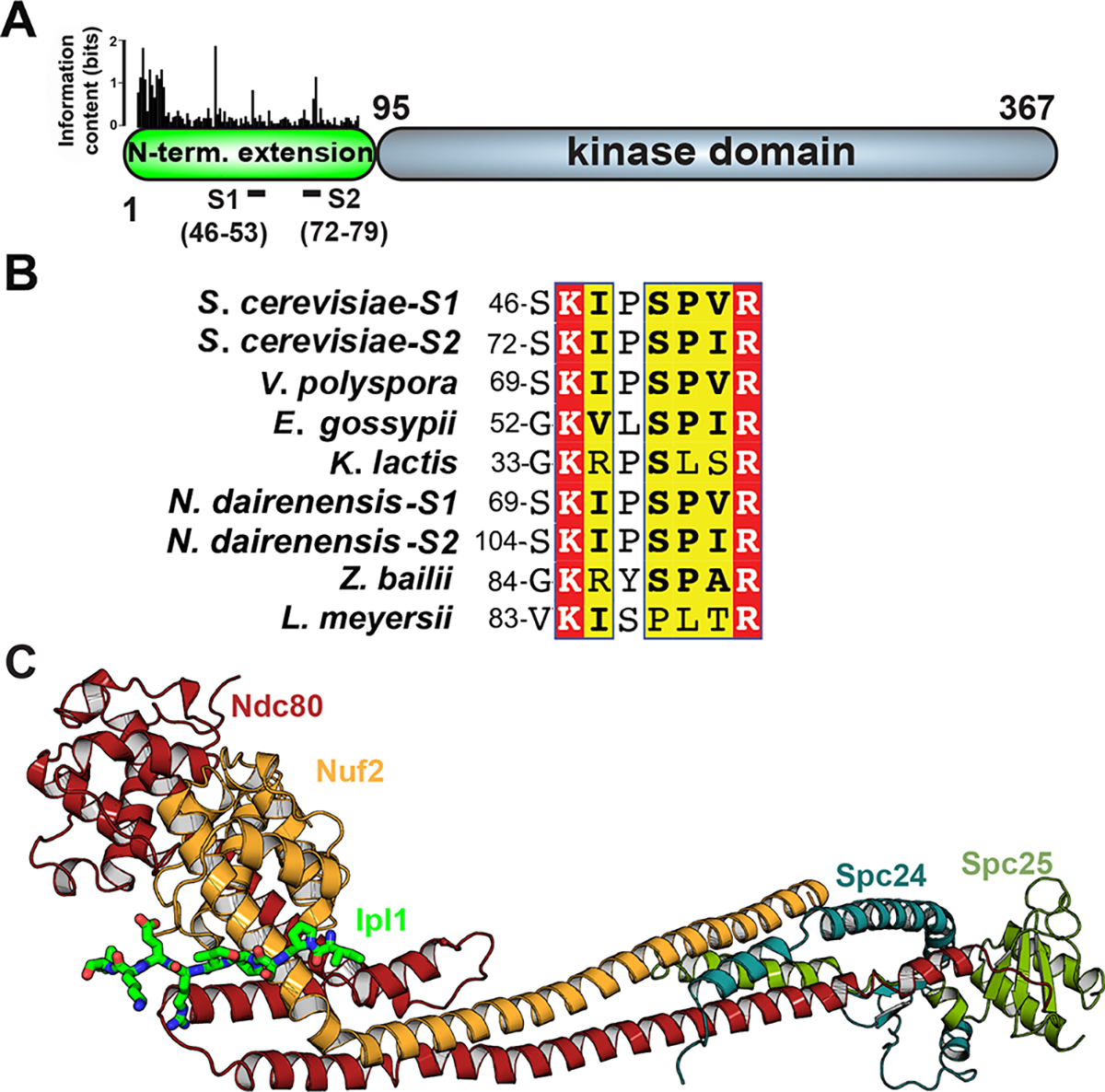
Crystal structure of a segment of the Ipl1 N-terminal domain that binds Ndc80c. **(A)** Schematic representation of the domain structure of Ipl1. “S1” and “S2” indicate each instance of a duplicated sequence that binds Ndc80c. **(B)** The sequences of S1 and S2 from **(A)** aligned with homologous segments of Ipl1 from other point-centromere yeast. **(C)** Cartoon representation of the crystal structure of Ndc80c^dwarf^ with Ipl1 S1 (residues 45–59) fused to the N-terminus of Nuf2. See also Figures S1, S2, S3, S5 and Table S1.

Ipl1 and Mps1 both phosphorylate residues in the N-terminal extension of Ndc80^7,8,34,53^. Documented Ipl1 kinetochore contacts are between its CPC partners, Sli15/INCENP and Bir1/survivin, and the inner-kinetochore COMA complex and the CBF3 component Ndc10, respectively^54–56^. In the CPC, a long, largely flexible segment (~600 residues) of Sli15/INCENP intervenes between the N-terminal region that interacts with Bir1 and Nbl1 and the segment that interacts with the Ipl1 kinase domain. There is also an approximately 100-residue, N-terminal extension of the Ipl1 kinase domain (Figure 5A). An AF2 prediction similar to the ones we carried out for Mps1 suggested that Ipl1 might also interact with Ndc80c at the site shared by Dam1 and Mps1 (Figure 2B). The predicted local distance difference test (pLDDT), which reports a per-residue confidence level, was somewhat lower than for the Mps1 (Figure 2A) and Dam1 (Figure 2C) predictions, but comparison of the predicted aligned error (pAE) plots suggests strong similarity with the second parts of the bipartitie motif present in the other two and weaker similarity with the first parts. The second-part binding motif, SKIPSP(V/I)R, is present with some variation, in Ipl1/AuroraB from other point-centromere yeast (Figure 5B). *S. cerevisiae* Ipl1 has two copies of this motif; the AF2 predictions split relatively evenly between the two alternatives. AF2 also yielded a consistent prediction for the somewhat divergent, single-copy motifs in *K. lactis* and *E. gossypii* (Fig S3).

We measured the Ipl1 peptide affinity, both directly (Figure 3A) and by measuring displacement of a fluorophore-labeled Mps1 peptide (Figure 3B) as a function of increasing concentrations of unlabeled Ipl1 peptide. We also carried out the converse experiment, measuring displacement of a fluorophore-labeled Ipl1 peptide as a function of unlabeled Mps1 peptide concentration (Figure 3D). The Ipl1 peptide bound more weakly than those from Dam1 and Mps1, but avidity contributed by the two copies of the conserved motif might enhance binding of the intact protein. The Mps1 peptide competed strongly with binding of the Ipl1 peptide, consistent with overlapping binding sites.

The Ipl1 motif that binds Ndc80c is also the target of phosphorylation by Cdk1, which preferentially modifies serine or threonine at sites with the sequence (S/T)Px(R/K)^57^. A Bim1 binding motif SxIP^58^ immediately precedes the SP(V/I)R Ndc80c-binding sequences in *S. cerevisiae* Ipl1 (Figure 5B), and phosphorylation of S50 and S76 appears to suppress Ipl1 association with Bim1 until activity of the Cdk1 kinase Cdc28 declines in anaphase and the CPC relocates to the spindle^57^. Although the Bim1-binding consensus is absent in many other point-centromere yeast (Figure 5A), the Cdk1 consensus is almost always present.

### Structure of Ndc80c chimera with Ipl1 binding motif

We generated a chimeric construct that fused to the N-terminus of Ndc80c^dwarf^ Nuf2 a peptide containing Ipl1 residues 26–59, with 40–44 (a disordered loop in the AF2 prediction) deleted,. The chimera crystallized in space group P43212 (a = b = 115Å, c = 424Å), with two complexes in the asymmetric unit related by an approximate non-crystallographic dyad. We recorded anisotropic diffraction data to a minimum Bragg spacing of 3.9Å, but intensities of reflections in the a* and b* directions fell below significance at Bragg spacings of ~6 Å. Despite the anisotropically ordered crystals, we determined phases by molecular replacement and calculated electron density maps, both with all the observed Fourier terms and with terms restricted to a nominal resolution of 6 Å (see Methods). When phased only on the molecular-replacement model, a difference map showed strong density for ordered parts of the Ipl1 segment and a structure overall that was consistent with the AF2 prediction between Ipl1 residues 48 and 56 (Figure 5, Figure 2B). The density for this segment was continuous with that for the Nuf2 subunit of the non-crystallographic-symmetry related complex (Figure S1B,C). Thus, the asymmetric unit is a “domain-swapped” dimer, and the interactions may have been essential for the crystals to form, suggesting that the interaction might occur in solution. Although both sequence alignment and AF2 prediction suggest a potential first part of a bipartite motif, the density map was not continuous enough in the predicted region to build any structure with confidence. Fig S2B shows a comparison of the AF2 prediction and the x-ray crystal structure.

### Comparison of Mps1, Ipl1 and Dam1 interactions with Ndc80c

Dam1 and Mps1 both bind the head of Ndc80c through segments in extended regions of their polypeptide chains. Comparison of the interactions of Dam1 and Mps1 with Ndc80c (Figure S4) and alignment of the respective binding motifs (Figure 3F) illustrates that the two are very similar. Moreover, the competition binding experiment shows that their affinities are nearly the same (Figure 3B). The bipartite consensus binding motif has, in both cases, a linker of variable length between the two parts. The first part of the motif includes two or more basic residues followed by two hydrophobic residues; the second part is a tetrapeptide terminating in an invariant arginine. The contacts on Ndc80c are likewise conserved. The basic residues at the N-terminal end of the bipartite motif pair with conserved glutamic-acid residues on Ndc80 (Glu 269, 272, 276, *S. cerevisiae* numbering); the specific pairing may vary from ortholog to ortholog, as both arginine and glutamic-acid side chains can adopt multiple configurations. The pair of hydrophobic residues fits into a pocket between Nuf2 helices A and G. The invariant arginine at the end of the motif pairs though a salt bridge with Asp295 (*S. cerevisiae* numbering), likewise invariant (or in a few cases, glutamic acid) among point-centromere yeast. In so doing, the arginine side chain reaches across and caps the C-terminus of Ndc80 helix H (for an alignment of Ndc80 sequences from ten diverse point-centromere yeast, see data_s3_alignment_ndc80_budding_yeasts in the Supplementary Information for^47^). The interaction of conserved Asn128 (*S. cerevisiae* numbering) with the main-chain hydrogen-bonding groups of the residue preceding the arginine may also extend to metazoans (Figure S5 and^38^). The conformations of the Mps1 and Dam1 peptides three residues to either side of the invariant arginine are the same, within the likely accuracy of the structures in that region and the presence of nearby crystal lattice contacts. Finally, a recent structure of Dam1c and Ndc80c bound together with a microtubule^59^ confirms that the Dam1-Ndc80c interaction, as seen in our published crystal structure^47^, is fully compatible with Ndc80c microtubule attachment; this compatibility is also consistent with the observation in the accompanying paper, that Mps1 can associate unhindered with microtubule-bound Ndc80c^40^

The Ipl1 consensus includes the essential features of the second part of the bipartite motif in Dam1 and Mps1. Conservation of a WRI motif (Figure 3F) in several other point-centromere yeast suggests a possible first part of a bipartite motif, with a variable spacing between the two parts (as in Dam1 and Mps1). In the AF2 prediction of Ipl1 bound with Ndc80:Nuf2 (Figure 2B), the tryptophan fills the same pocket occupied by the pair of hydrophobic residues in the other two complexes. The density maps of the Ipl1 chimera, however, even when restricted to Bragg spacings greater than 6 Å, did not show well defined and connected density for polypeptide chain N-terminal to Ile 48. The refined conformation of the peptide between residues 50 and 56 (or 76 and 82) is very similar to that of the Mps1 motif, and hence also to the Dam1 peptide, even though the starting point for fitting the peptide was independent of the Mps1 and Dam1 structures.

## DISCUSSION

The results presented above show that peptide segments in the N-terminal extensions of both Mps1 and Ipl1 bind a site on the head of Ndc80c that is essentially the same as the site bound by a segment in the C-terminal extension of Dam1. Ndc80c connects the centromere-proximal components of a kinetochore with the MTs of the mitotic spindle, and the DASH/Dam1c coordinates multiple Ndc80 complexes, thereby enabling end-on kinetochore-MT attachment. The Ndc80c head is thus a communication hub for ensuring a robust linkage between centromere and spindle and for integrating the SAC with tension sensing and error correction.

Mps1 is the sole co-purifying kinase on isolated, unattached kinetochores^17,34^. We infer that it is probably bound with Ndc80c through the contact described here, although there could be additional, so far undetected, interactions with segments elsewhere in the long N-terminal extension. Side-on attachment of a kinetochore to a spindle microtubule need not displace Mps1, and even end-on attachment, which requires DASH/Dam1c, need not do so, as Dam1c/DASH interaction with Ndc80c includes contacts between the coiled-coil shaft of Ndc80 and both Ask1 and Spc19/Spc34. Moreover, there are approximately 8 copies of Ndc80c in prometaphase/metaphase^32^, and 16–17 copies of Dam1 in a single DASH/Dam1c ring^31,59^. Thus, mixed states are likely, in which Mps1 continues to be present (at decreasing levels) and hence continues to activate the SAC, until fully displaced by (dephosphorylated) Dam1. Autophosphorylation of Mps1 appears to result in its release (in vitro) from kinetochore association^39^, but the relevant sites have not been determined. There are no serines or threonines in conserved positions within the bipartite motif, ruling out a direct regulation by phosphorylation of the Ndc80c interacting segment analyzed here.

In human Mps1, two distinct regions have been implicated in kinetochore interaction: a short N-terminal segment preceding a three-repeat TPR domain^4^, features absent in yeast Mps1, and a conserved segment in the long “middle region” that links the TPR motifs with the kinase domain at the C-terminus^38^. Mutation of residues on the surface of the Ndc80 CH domain have been reported to affect binding of the former; mutation of N126 (corresponding to N128 in *S. cerevisiae*) to alanine interferes with binding of the latter^38^. The sequence of the conserved, Mps1 middle-region segment includes a QSCPFGR motif, with invariant arginine in various vertebrate species; AF2 yields very high confidence association of that segment (and several residues C-terminal to it) at the site in the human Ndc80c head corresponding precisely to the communication hub site we have characterized in yeast (Figure S5A). The specific Mps1 interaction with Nuf2 seen in yeast thus appears also to be present in metazoans.

Several lines of evidence suggest that Mps1 may under some circumstances recruit DASH/Dam1c to kinetochores, by modification of residues on Dam1 or on another component of the heterodecameric complex^48,60^. Principal candidates are Dam1 residues Ser218 and Ser 221, which are part of a conserved motif in point-centromere yeast Dam1 (218-S/TxASFVxNP-226, *S. cerevisiae* numbering, preceded by several negatively charged residues)^61^. Available structural information does not provide hypotheses about possible binding partners or about autoinhibition mechanisms for ring formation that might involve this short, conserved motif, although conservation suggests a defined function. The other reported Mps1 site in Dam1, Thr15, is part of the N-terminal “staple” that connects one DASH protomer with another^59^; phosphorylation of Thr15 by Mps1, like that of Ser20 by Ipl1, might therefore disfavor ring formation, rather than stabilize it^62^.

Ipl1/Aurora B has at least two distinct roles in regulating kinetochore assembly and attachment. Phosphorylation of Dsn1 activates the MIND/Mis12 complex by relieving autoinhibition and allowing binding of Mif2/CENP-C and Ame1 to sites on the Mtw1-Nnf1 globular “head^63–66^. Subsequently, the kinase mediates error correction and reverses end-on attachment in the absence of tension by phosphorylating both the Ndc80 N-terminal extension and at least three components of DASH/Dam1c -- Dam1 itself, Ask1, and Spc34^33^ -- or the Ska complex in metazoans^67^. In yeast, binding of Bir1 with centromere-bound Ndc10 can recruit Ipl1 early in kinetochore assembly, which begins immediately after replication of yeast centromeres at the beginning of S phase. Binding of Sli15 with COMA may provide a further anchor point, once the Ctf19 complex (Ctf19c) has assembled on the Cse4 nucleosome^54,55^. Tethering of Ipl1, through Sli15 and Bir1, to centromere-bound Ndc10 could compensate for the relatively weak affinity of the Ipl1 motif for the Ndc80c head, and the interaction with the Ndc80c head could then localize the kinase domain close to its targets. The Bir1 interaction with Ndc10 and our proposed Ipl1 contact with the Ndc80c head could co-exist: about 600 Sli15 residues intervene between its attachments to Bir1 and Ipl1, enough to stretch between CDEIII, to which Ndc10 binds, and the head of Ndc80. Bir1 tethering to Ndc10 appears not be be essential, however, as deletion of the Bir1-binding element at the N-terminus of Sli15 does not compromise accurate chromosome segregation^45^. An additional interaction with Ctf19^54,55^, as well as the contact with Ndc80c detected here, could account for the lack of defective phenotype in the Sli15 truncation mutants. Whether Ndc80c head binding by the N-terminal extension of Ipl1 can occur concomitantly with the proposed Sli15-COMA interaction will depend on the position, not yet determined, of the relevant residues on Sli15. In the absence of tension, the proximity of the Ipl1 kinase domain afforded by interaction between the Ndc80c head and the N-terminal extension might be important for capturing target sites on the Ndc80 N-terminal extension when some or all of that extension dissociates from binding with a microtubule.

Human Aurora B also has a candidate Ndc80c binding motif (TLPQR) in the 70-residue N-terminal extension that precedes the kinase domain in the polypeptide chain. AF2 predicts with high confidence an association very similar to the predicted Mps1 association, including participation of several residues C-terminal to the key arginine (Figure S5B). Intermolecular interactions of metazoan Mps1 and Aurora B with the Ndc80c head that are very similar to those found in yeast suggest conservation of the Ndc80c site as a communication hub.

Detection of a segment in the Ipl1 N-terminal extension that can interact with the same site on the Ndc80c head that binds Dam1 and Mps1 and observations that Ipl1 and Mps1 have partly overlapping roles in error correction raise the question of when, in the interval from S-phase to late prometaphase, does each of these kinases have its most important function and what are its critical targets. Ipl1 appears to be required for error correction throughout prometaphase, even after formation of two distinct kinetochore clusters, which can still contain syntelic attachments^21^. Mps1 must likewise be present, at least on any pair not under tension, to keep the SAC engaged. Future experiments will need to determine the trade-off among Mps1, Ipl1, and Dam1 at the Ndc80c communication hub, the respective phosphorylation targets when Mps1 or Ipl1 binds there, and the importance of mixed states, with some of the Ndc80 complexes on any kinetochore bearing one of the kinases, some bearing the other, and even some bound with Dam1.

### RESOURCE AVAILABILITY

#### Lead contact

Further information and requests for resources and reagents should be directed to the lead contact, Stephen C. Harrison (harrison@crystal.harvard.edu).

#### Materials availability

Expression constructs are available and should be requested from the lead contact.

#### Data and code availability

- Data needed to evaluate the conclusions in the paper are in the main figures and/or supplemental information. Structure factors and coordinates have been deposited in the Protein Data Bank (https://www.rcsb.org/), with entry numbers 8V10 (Mps1-Ndc80^dwarf^ chimera) and 8V11 (Ipl1- Ndc80^dwarf^ chimera); they are publicly available as of the date of publication.
- The paper does not report original code
- Any additional information required to reanalyze the data reported in this paper is available from the lead contact upon request.

### EXPERIMENTAL MODEL AND SUBJECT DETAILS

Products of ligation reactions were used to transform *E. coli* DH5α cells (New England Biolabs); single colonies were grown overnight at 37°C in 2XYT medium and plasmids purified using a Monarch miniprep kit (New England Biolabs).

Proteins were co-expressed in *E. coli* BL21 T7-express (New England Biolabs). Cells were grown at 37° in 2XYT medium to an OD600 of 0.8, induced with 500 μM IPTG, and incubated overnight with shaking at 18°C.

### METHODS DETAILS

#### Cloning and protein expression constructs

Constructs were the same as those used previously^51^, except for the exclusion of a non-native sequence N-terminal to Nuf2 that promoted crystallization in the earlier study and the addition of Mps1 and Ipl1 fragments N-terminal to Nuf2, as described below. Cloning of the Mps1:Nuf2 and Ipl1:Nuf2 fusions, and the Nuf2 N128A and F8P9AA mutants was carried out by digesting pETDuet1 containing inserts coding for dwarf versions of Ndc80 and Nuf2 with NdeI and XhoI (New England Biolabs) to remove the existing Nuf2 insert. The digested vector was excised from an agarose gel, purified using a gel extraction kit (Qiagen) and treated with Quick CIP (New England Biolabs). Synthetic genes coding for Mps1:Nuf2, Ipl1:Nuf2, N128A Nuf2 and F8P9AA Nuf2 were digested with NdeI and XhoI and purified using a Min-Elute enzyme reaction cleanup kit (Qiagen). Ligations were carried out using a Quick Ligation Kit (New England Biolabs) with a 1:3 molar ratio of vector:insert; 3 μL of the ligation reactions were used to transform *E. coli* DH5α cells (New England Biolabs). Single colonies were grown overnight at 37°C and plasmids purified using a Monarch miniprep kit (New England Biolabs). The constructs used to express GST-fusions of Mps1 fragments were ordered pre-cloned into a pGEX-6P1 vector (Genscript).

#### Protein expression

Proteins were co-expressed in *E. coli* BL21 T7-express (New England Biolabs). Cells were grown at 37° in 2XYT medium to an OD600 of 0.8, induced with 500 μM IPTG, and incubated overnight with shaking at 18°C. Cell pellets from 6 L of culture were resuspended in 100 mL of buffer containing 100 mM Tris pH 8.0, 250 mM NaCl, 10 mM imidazole pH 8.0, 2 mM tris(2-carboxyethyl)phosphine (TCEP), 1 mM PMSF, 2 “Complete” protease inhibitor tablets (Roche), 25 μg/mL lysozyme (Gold Biochem) and 5 μg/mL DNaseI (Gold Biochem). Cells were lysed by sonication (flat tip probe, 50% duty cycle for a total sonication time of 1 minute) and the lysate clarified by centrifugation (20,000 rpm using a Beckmann Avanti centrifuge equipped with JA-20 rotor) and applied to (5 mL) Ni-NTA agarose (Pierce) equilibrated with 20 mM Tris pH 8.0, 100 mM NaCl, 10 mM imidazole pH 8.0, 2 mM TCEP (equilibration buffer). Beads were washed with (50 mL) equilibration buffer adjusted to 20 mM imidazole and 500 mM NaCl and then with (100 mL) equilibration buffer. Proteins were eluted with equilibration buffer adjusted to 400 mM imidazole pH 8.0 and 100 mM NaCl. Eluates were treated overnight with TEV protease (purified in-house) to remove the 6-His tag from the N-terminus of Ndc80 (except for protein to be immobilized for pulldown experiments) and subjected to anion exchange chromatography on a 5mL Hi-trap Q HP (Cytiva). Elution was carried out in a buffer comprising 20 mM Tris pH 8.0 and 2 mM TCEP with a gradient from 30 mM to 1M NaCl over 15 column volumes, followed by size-exclusion chromatography using a Hi-load Superdex 200 16/60 column (Cytiva). For protein crystallization the column was equilibrated with 10 mM HEPES pH 7.2, 100 mM NaCl, and 2 mM TCEP. For fluorescence polarization binding and pulldown experiments, the buffer was 10 mM HEPES pH 7.2, 150 mM NaCl, 2 mM TCEP.

The Mps1 fragments used for pulldown experiments were ordered from Genscript and cloned into pGEX6P1 (Novagen) to express them as GST fusions. Cells were grown at 37°C in 2XYT media to an OD600 of 0.8, induced with 500 μM IPTG, and incubated overnight with shaking at 225 rpm at 18°C. Cell pellets from 2 L of culture were resuspended in 30 mL of buffer containing 100 mM Tris pH 8.0, 250 mM NaCl, 2 mM TCEP, 1 mM PMSF, 25 μg/mL lysozyme (Gold Biochem), 5 μg/mL DNaseI (Gold Biochem) and a single “Complete” protease inhibitor tablet (Roche). Cells were lysed by sonication and the lysate clarified by centrifugation and applied to glutathione agarose (1 mL bed volume) (Pierce). The resin was washed with 20 mM Hepes pH 7.2, 250 mM NaCl, and 2 mM TCEP (wash buffer). Protein was eluted from the resin by applying 6 mL of wash buffer supplemented with 20 mM reduced glutathione (Sigma).

#### Pulldown Experiments

For the pulldown experiments in Figure 1, Ndc80c^dwarf^ with a 6-His affinity tag on the N-terminus of Ndc80 was immobilized to saturation on Ni-NTA agarose by incubation in a buffer containing 20 mM Hepes pH 7.2, 150 mM NaCl and 2 mM TCEP with gentle agitation at 4°C. Beads were pelleted by centrifugation in a mini benchtop centrifuge, washed three times with 20 mM HEPES pH 7.2, 150 mM NaCl, 2 mM imidazole pH 7.2, 2 mM TCEP, and incubated with the Mps1-GST fusion eluted from the glutathione agarose for 30 min. Beads were again pelleted, washed three times with wash buffer, pelleted a final time, and the wash buffer aspirated from the tube. To elute bound proteins, 50 μL of wash buffer supplemented with 500 mM imidazole pH 7.2 was added to ~25 μL of beads. After an additional spin to remove the beads, the eluted proteins were visualized by SDS-PAGE.

#### Peptide binding experiments

Mps1 (137–171), Ipl1 (26–39;45–59), and Dam1 (254–270;290–305) peptides with added C-terminal cysteines, were synthesized by the Tufts University Core Facility. Peptides were labelled with Bodipy FL maleimide (Thermo-Fisher) according to the manufacturer’s instructions. The labeled peptide was separated from unreacted dye by cation exchange chromatography with Source 15S resin (Cytiva). Fluorescence polarization was measured at 25°C with an Envision plate reader (Perkin Elmer) equipped with a filter set optimized for fluorescence polarization of FITC, which has the same absorption and emission spectra as those of Bodipy FL. For direct binding experiments, an 860 μM solution of Ndc80c^dwarf^ in a buffer containing 20 mM Hepes pH 7.2, 150 mM NaCl, 2 mM TCEP was subjected to 2-fold serial dilutions and subsequently supplemented with an equal volume of a buffer-matched solution of 40 nM Bodipy-FL labelled Mps1 or Ipl1 peptide. Fluorescence polarization was measured in triplicate. For competition binding experiments, 2 mM solutions of each peptide, dissolved in 20 mM Tris pH 7.2, 100 mM NaCl, 2 mM TCEP, were subjected to serial 2-fold dilutions. An equal volume of a buffer-matched solution containing 40 nM Bodipy-FL labelled peptide and 1 μM Ndc80c^dwarf^ was added to each of the dilutions, and fluorescence polarization was measured in triplicate.

#### AF2 predictions

We used version 2.2.2 of Alphafold_multimer, as curated by SBGrid^69^, for all AF2 predictions in this work^52^. The predicted aligned error (pAE) plots were generated from the AF2 .pkl files by AlphaPickle^70^. Structures and pAE plots are for the ranked_0 model in all figures shown. We verified in each case that all highly ranked models were essentially the same for all residues with pLDDT>50.

#### Ndc80c^dwarf^-Mps1 chimera crystallization and structure determination

Ndc80c^dwarf^, with residues 137–171 of *S. cerevisiae* Mps1 appended to the N-terminus of Nuf2, was concentrated, by centrifugation in an Amicon centrifugal concentrator with a 30 kDa molecular weight cutoff, to 25 mg/mL in 10 mM Hepes pH 7.2, 100 mM NaCl, 2 mM TCEP. Crystals were grown by vapor diffusion in hanging drop format in 24-well plates (Hampton Research). Crystals used for diffraction data collection grew from drops in which 1 μL of protein solution was mixed with 1 μL of a well solution containing 13% polyethylene glycol 8000, 1M sodium chloride, 100 mM PIPES pH 6.1. Crystals were harvested, soaked for 1–2 minutes in a well solution supplemented with 25% glycerol, and immersed immediately in liquid nitrogen. The complex crystallized in space group C222_1_ (a = 72.52 Å, b = 168.45 Å, c = 229.57 Å). Data to a minimum Bragg spacing of 3.29 Å were recorded on beamline 201 at the Advanced Light Source (Lawrence Berkeley Laboratory, Berkeley CA) and indexed and integrated using DIALS, as implemented in xia2^71^. The data were scaled and merged using the STARANISO server^72^. The structure was determined by molecular replacement in Phenix^73^, using three non-overlapping segments of Ndc80c^dwarf^ (PDB 5TCS) as search models. The molecular replacement maps showed clear density for the Mps1-derived peptide. Model building was carried out in Coot^74^ and refinement, in Phenix^73^. Data statistics are in Table S1.

#### Ndc80c^dwarf^-Ipl1 chimera crystallization and structure determination

Ndc80c^dwarf^, with residues 26–39;45–59 of *S. cerevisiae* Ipl1 appended to the N-terminus of Nuf2 was concentrated to 40 mg/mL in 10 mM Hepes pH 7.2, 100 mM sodium chloride, 2 mM TCEP. Crystallization screens were set up in sitting drop format using a Mosquito liquid handling robot. The 400 nL drops contained equal volumes of protein and well solution. Crystals grew from drops equilibrated with 15% polyethylene glycol 2000 monomethyl ether, 500 mM sodium chloride, 50 mM Tris pH 7.5. Crystals were harvested directly from the screening drop, soaked for 1–2 minutes in a well solution supplemented with 25% glycerol, and immersed immediately in liquid nitrogen. The complex crystallized in space group P4_3_2_1_2 (a = 114.81 Å, b = 114.81 Å, c = 424.17 Å). Data were recorded on beamline 201 at the Advanced Light Source and integrated with XDS^75^. Substantial anisotropy in the diffraction required scaling and mering using the STARANISO server^72^. The minimum Bragg spacing following anisotropic scaling was 3.93 Å in the “best” direction and 6.76 Å in the “worst”. We determined the structure by molecular replacement in Phenix^73^, using three search models derived from Ndc80c^dwarf^ (PDB 5TCS). There were two complexes in the asymmetric unit. The 2Fo-Fc and Fo-Fc molecular replacement maps had clear density for the appended Ipl1 peptide in both non-crystallographic symmetry (ncs) related copies. The density also showed that the peptide appended to one ncs copy of Ndc80c^dwarf^ was bound to the ncs-related copy -- i.e., that the two ncs-related complexes are a “domain-swapped” pair (Figure S2B,C). We used the program O^76^ to fit a peptide comprising residues 48–56 of Ipl1 into the Fo-Fc density, truncated to 6 Å in all directions, using the AF2 model as a starting point. The density bridging from the C-terminus of this peptide to the N-terminus of the ncs-related Nuf2 had the dimensions of a short α helix. A poly-alanine helix of the correct length for the chimeric construct and built into that density just spanned the required endpoints (Figure S2B,C). We did not add the (known) side chains beyond the β carbons. Refinement was carried out in Phenix^73^. Data statistics are in Table S1.

### QUANTIFICATION AND STATISTICAL ANALYSIS

Binding and inhibition curves in Figure 3 were fit in Graphpad Prism (https://www.graphpad.com) with a four parameter logistic curve regression model. The uncertainties quoted are from the 95% confidence intervals reported by the program.

## Supplementary Material

1

## Figures and Tables

**Key resources table:** 

REAGENT or RESOURCE	SOURCE	IDENTIFIER
**Bacterial and virus strains**		
T7-express *E. Coli*	New England Biolabs	C2566H
DH5α *E. Coli*	New England Biolabs	C2987H
**Chemicals, peptides, and recombinant proteins**		
TCEP	Gold Biochem	TCEP25
IPTG	Gold Biochem	I2481C100
PMSF	Gold Biochem	P470-10
cOmplete^™^, Mini, EDTA-free Protease Inhibitor Cocktail	Roche	1183617000
PEG 8000	Sigma Aldrich	89510
Trizma Base	Sigma Aldrich	93362
Glutathione agarose	Pierce	16101
Ni-NTA agarose	Qiagen	30230
XhoI	New England Biolabs	R0146S
NdeI	New England Biolabs	R0111S
DnaseI	Gold Biochem	D-301-100
Lysozyme	Gold Biochem	L-040-1
Glutathione agarose	Pierce	16101
Ni-NTA agarose	Qiagen	30230
2XYT media	RPI Research Products	X15680-5000.0
Ipl1 26–59 Δ40–44 TSRINKPWRISHSPNSKIPSPVREKLNRLC	Tufts Core Facility	N/A
Mps1 137–171 RQNMKEDITAKYAERRSKRFLISNRTTKLGPAKRAC	Tufts Core Facility	N/A
Dam1 254–306 Δ271–289 RRKSILHTIRNSIASGARISLGSGAARVVNGPVTC	Tufts Core Facility	N/A
Mps1 R151D R152D R155D RQNMKEDITAKYAEDDSKDFLISNRTTKLGPAKRAC	Tufts Core Facility	N/A
Mps1 F156D L157A RQNMKEDITAKYAERRSKRDAISNRTTKLGPAKRAC	Tufts Core Facility	N/A
Mps1 R170S RQNMKEDITAKYAERRSKRFLISNRTTKLGPAKSAC	Tufts Core Facility	N/A
Mps1 F156D L157A R170S RQNMKEDITAKYAERRSKRDAISNRTTKLGPAKSAC	Tufts Core Facility	N/A
BODIPY^™^ FL Maleimide	Invitrogen	B10250
**Deposited data**		
Mps1:Ndc80c chimera coordinates	Protein Data Bank	8V10
Ipl1:Ndc80c chimera coordinates	Protein Data Bank	8V11
Ndc80c(dwarf) coordinates	Protein Data Bank	5TCS
**Oligonucleotides**		
**Mps1:Nuf2** ACCTGTACTTCCAATCCAATCATATGCGTCAGAACA TGAAGGAAGACATTACGGCAAAGTACGCCGAGCGC CGTTCCAAACGCTTTTTAATCTCAAATCGCACTACG AAACTGGGGCCGGCCAAGCGCGCTTCTCGCAACCA GGATGTCTTCCCCATCCTTGACCTGCAAGAATTAGT GATCTGCCTTCAGTCCTGTGACTTTGCCCTTGCAAC CCAAGAGAACATCAGCCGTCCAACTTCTGACTACAT GGTCACACTTTACAAACAGATTATTGAGAATTTTATG GGGATTAGCGTAGAGTCTCTGTTGAACTCTTCGAAC CAAGAGACCGGTGATGGGCATCTGCAAGAAGAAAA CGAAAATATCTACTTAGACACGCTTAACGTACTTGTA TTGAATAAAATTTGTTTCAAGTTCTTTGAGAACATTG GTGTGCAAGACTTCAACATGACTGACCTGTACAAAC CCGAAGCCCAACGCACTCAACGCCTGCTGTCTGCA GTCGTGAATTACGCACGTTTCCGCGAAGAACGCAT GTTCGATTGCAACTCCTTTATTTTACAGATGGAATCG TTGCTGGGTCAAATCAACAAATTAAACGATGAAATTA AGCAGCTTCAGAAAGATTTCGAAGTGGAAGTCAAAG AAAT	Integrated DNA Technologies	N/A
**Ipl1:Nuf2** ACCTGTACTTCCAATCCAATCATATGACTAGCCGTA TCAATAAACCTTGGCGTATATCTCATTCCCCCAATA GCAAGATACCGTCGCCCGTCAGAGAGAAACTTAAC AGACTGTCGCGTAACCAAGATGTATTTCCTATATTG GACTTGCAGGAGCTTGTCATCTGCCTTCAATCATGT GATTTTGCCTTGGCTACCCAAGAAAACATCAGTCGC CCGACTTCCGATTACATGGTAACTCTTTATAAGCAG ATCATAGAAAATTTCATGGGCATTAGTGTCGAGTCA TTCCTGAACTCTTCGAACCAGGAAACAGGGGACGG GCACCTGCAAGAGGAGAACGAGAATATCTATCTGG ATACCTTAAACGTGTTGGTTCTTAATAAAATCTGCTT TAAATTCTTCGAAAACATCGGGGTCCAAGATTTTAA CATGACGGACTTGTATAAACCCGAAGCACAACGTAC TCAAAGACTGCTGTCTGCCGTAGTGAATTATGCGCG TTTCCGCGAGGAACGCATGTTTGACTGTAATTCTTTT ATATTGCAGATGGAGTCCTTACTTGGACAGATTAAC AAATTGAATGATGAAATAAAACAATTACAAAAGGACT TCGAGGTCGAGGTTAAAGAAATAGAGATAGAATATA GCTTATTGAGTGGTCACATCAACAAATACATGAACG AAATGTTAGAATACATGCAATAATGACTCGAGATTG GAAGTGGATAACGGAT	Integrated DNA Technologies	N/A
**Nuf2(N128A)** ACCTGTACTTCCAATCCAATCATATGAGTCGTAATCA GGACGTGTTTCCGATTCTGGACCTTCAGGAACTGGT TATTTGTTTGCAGAGCTGTGATTTCGCCTTAGCTAC GCAGGAGAATATCAGCCGCCCAACGTCAGACTACA TGGTTACGTTATACAAGCAAATTATCGAGAACTTTAT GGGAATCTCGGTGGAGTCGCTTTTGAACTCCAGCA ATCAGGAGACAGGCGATGGACACTTACAGGAGGAG AATGAAAACATTTATCTTGATACATTGAACGTGCTGG TGCTGAATAAGATTTGTTTTAAGTTTTTTGAGAACAT CGGGGTTCAGGACTTTAACATGACCGATTTATACAA ACCGGAAGCACAACGCACGCAACGCTTGCTTTCCG CTGTAGTAGCCTATGCGCGCTTCCGTGAAGAACGC ATGTTTGATTGCAACTCGTTTATCCTTCAAATGGAGT CATTATTGGGTCAAATCAACAAACTGAACGATGAGA TTAAGCAACTTCAAAAGGATTTTGAGGTGGAAGTCA AGGAAATCGAAATCGAGTACAGCCTTCTGAGCGGA CACATCAATAAGTACATGAACGAAATGCTGGAGTAT ATGCAGTGATGACTCGAGATTGGAAGTGGATAACG GA	Integrated DNA Technologies	N/A
**Nuf2(F8A P9A)** ACCTGTACTTCCAATCCAATCATATGAGCCGCAACC AAGACGTGGCAGCGATCTTAGACCTGCAGGAGTTG GTTATCTGTCTGCAGTCTTGCGACTTTGCGCTGGCT ACACAAGAGAACATTTCGCGCCCCACTTCGGATTAT ATGGTGACTTTGTACAAACAGATCATCGAAAATTTTA TGGGAATTTCCGTAGAGTCGTTGTTGAACTCTTCGA ACCAGGAAACAGGTGACGGGCATCTTCAAGAAGAA AACGAAAATATCTACTTAGATACTCTGAACGTCTTG GTACTGAACAAGATTTGCTTTAAATTTTTTGAGAACA TCGGAGTTCAGGACTTCAACATGACTGATTTGTATA AACCTGAGGCGCAACGCACCCAACGTTTGCTTTCA GCCGTGGTGAATTATGCGCGCTTTCGTGAGGAGCG CATGTTTGACTGCAATAGCTTTATCTTGCAAATGGA GTCACTGCTGGGCCAGATTAATAAATTGAATGACGA AATCAAGCAGTTGCAGAAGGACTTTGAGGTCGAAGT CAAAGAGATCGAGATCGAATACTCCTTATTGAGCGG TCACATTAATAAGTACATGAATGAAATGTTGGAGTAT ATGCAGTGATGACTCGAGATTGGAAGTGGATAACG GAT	Integrated DNA Technologies	N/A
**Mps1 22–46** GGATCCGGAGGCTCTGGAGGTTCAGGGGGAAGTG GGGGCTCGAGCGATGACGAGGAGTTCACCACCCC GCCGAAACTGTCTAACTTTGGTTCCGCGTTGCTGAG CCACACCGAAAAGACGAGCTAATGAGCGGCCGC	Novagen	N/A
**Mps1 151–171** GGATCCGGAGGCTCTGGGGGAAGTGGAGGTTCAG GGGGCTCCGAGCGCCGTAGCAAACGTTTCTTGATC AGCAACCGTACCACGAAGCTGGGTCCGGCGAAGC GCTAATGAGCGGCCGC	Novagen	N/A
**Mps1 217–267** GGATCCGGGGGAAGTGGCGGATCTGGAGGTTCAG GGGGCAGCGACTACGACAGCATTGATTTTGGTGAT TTGAATCCGATTCAATATATCAAGAAGCACAACCTG CCGACCTCCGACTTACCGCTGATCAGCCAGATCTA CTTCGATAAACAACGTGAAGAGAACCGCCAGGCGG CTCTGCGTAAACATTCGTCTTAATGAGCGGCCGC	Novagen	N/A
**Mps1 354–424** GGATCCGGAGGCTCTGGAGGTTCAGGGGGAAGTG GGGGTTCGAGCGAAAAGCGCGAAGTGCTGCGTAAT ATCAGCATTAACGCGAACCACGCCGATAATTTGCTG CAGCAAGAGAACAAGCGCCTGAAACGTTCTCTGGA CGACGCGATTACCAATGAAAACATCAACAGCAAGAA CTTGGAGGTTTTCTACCATCGTCCGGCACCGAAACC GCCAGTTACGAAAAAGGTGGAGATCGTCGAGCCGG CTAAATCCTAATGAGCGGCCGCC	Novagen	N/A
**Recombinant DNA**		
pETduet1	Novagen	71146
pRSFduet	Novagen	71341
**Software and algorithms**		
Alfafold_multimer version 2.2.2	SBGrid https://sbgrid.org/	Jumper et al.,2021^52^
AlphaPickle	https://github.com/mattarnoldbio/alphapickle	Arnold, 2021^70^
Graphpad Prism	https://www.graphpad.com/guides/prism	https://www.graphpad.com/guides/prism/latest/curve-fitting/reg_example_ria.html
xia2	SBGrid https://sbgrid.org/	Winter, 2010^71^
STARANISO server	Global Phasing https://staraniso.globalphasing.org/cgi-bin/staraniso.cgi	Tickle et al., 2022^72^
Phenix	SBGrid https://sbgrid.org/	Adams et al, 2010^73^
Coot	SBGrid https://sbgrid.org/	Emsley et al, 2010^74^
XDS	SBGrid https://sbgrid.org/	Kabsch, 2010^75^
O	SBGrid https://sbgrid.org/	Jones et al, 1991^76^
Skylign	skylign.org	Schuster-Böckler et al, 2004^68^
**Other**		
Quick CIP	New England Biolabs	M0525S
Quick ligation kit	New England Biolabs	M2200S
Monarch Mini prep kit	New England Biolabs	C2987H
QIAquick Gel Extraction Kit	Qiagen	28704
MinElute Reaction cleanup kit	Qiagen	28204
VDXm^™^ Plate with sealant	Hampton Research	HR3-306
EnVision Multimode plate reader	Perkin Elmer	2101-0010
Mosquito crystallization robot	TTP Labtech	N/A

## Highlights

The kinase Mps1 binds the Ndc80 complex through a conserved motif.

X-ray crystallography shows a common Ndc80c binding site for Mps1 and Dam1.

The Ipl1 N-terminal extension recognizes the Ndc80c site bound by Mps1 and Dam1.

Alphafold predictions suggest broad conservation of these shared interactions.
