# Supplementary material for: A communication hub for phosphoregulation of kinetochore-microtubule attachment
Source: Curr Biol. Author manuscript; Available in PMC 2025 Feb 23. (PMC11847324; doi:10.1016/j.cub.2024.04.067)
Supplement: 1 [file NIHMS2054243-supplement-1.pdf]

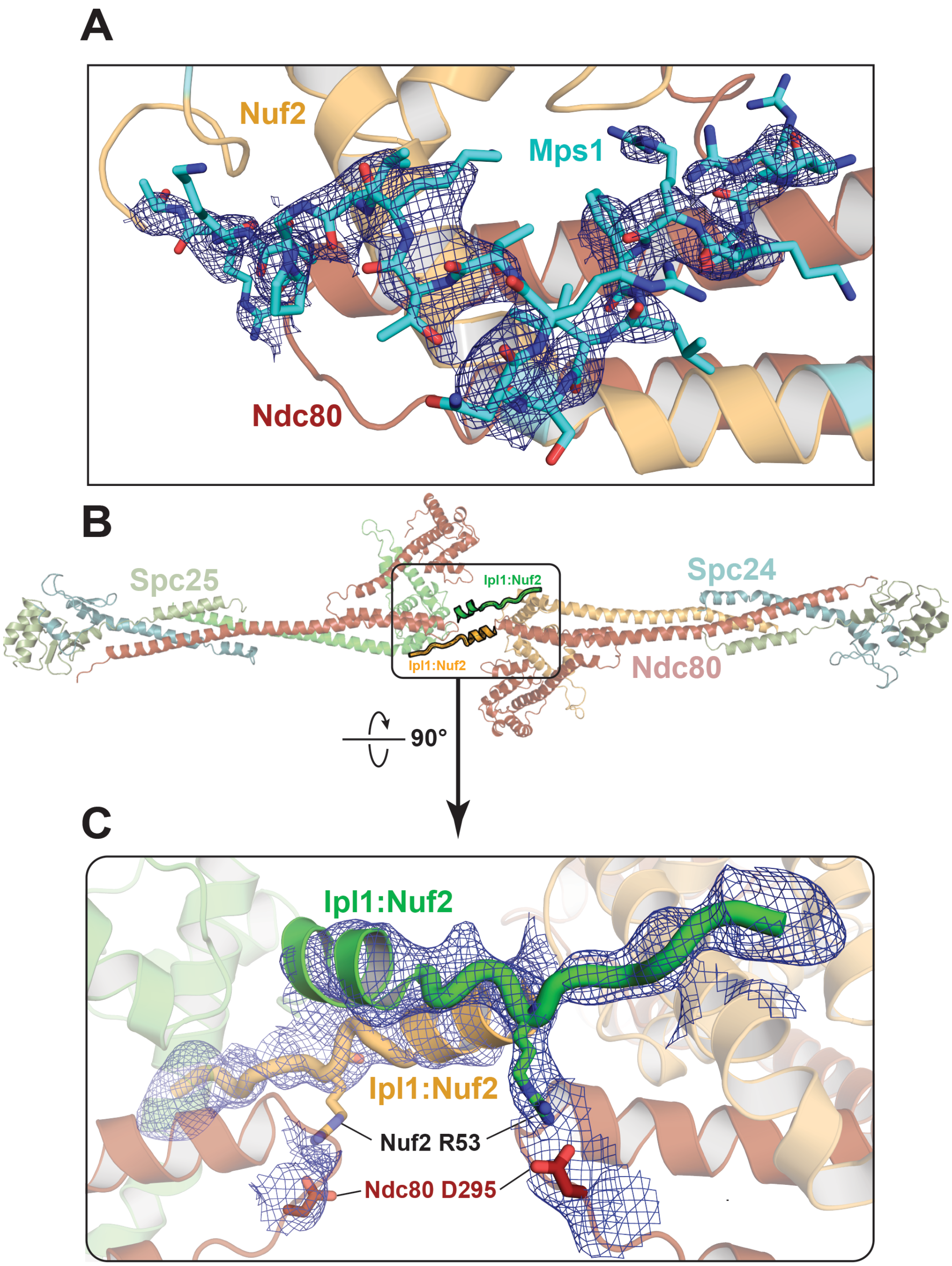

Figure S1. **Electron density superpositions. Related to Figures 4 and 5.**
(**A**) Detail of the Ndc80c^{dwarf} - Mps1 structure, showing 2Fo-Fc density, contoured at 1.0σ, around the Mps1 peptide. (**B**) Domain swap in the crystal structure of Ipl1 chimera with Ndc80c^{dwarf}. The Ipl1 peptide fused to the N-terminus of Nuf2 in one complex associates with the Ndc80:Nuf2 head in the other. Ndc80 in red; Nuf2 in orange for complex on the right and in green for the complex on the left. (**C**) Magnification of the boxed region of the three-dimensional structure in (**B**), viewed at 90° (as if from above (**B**)), with the 2Fo-Fc map over part of the structure, contoured at 1.0σ. Despite the restricted resolution, a tube of density represents the strong salt bridge between Arg 155 (invariant arginine in the binding motif) and Ndc80 Asp 295.

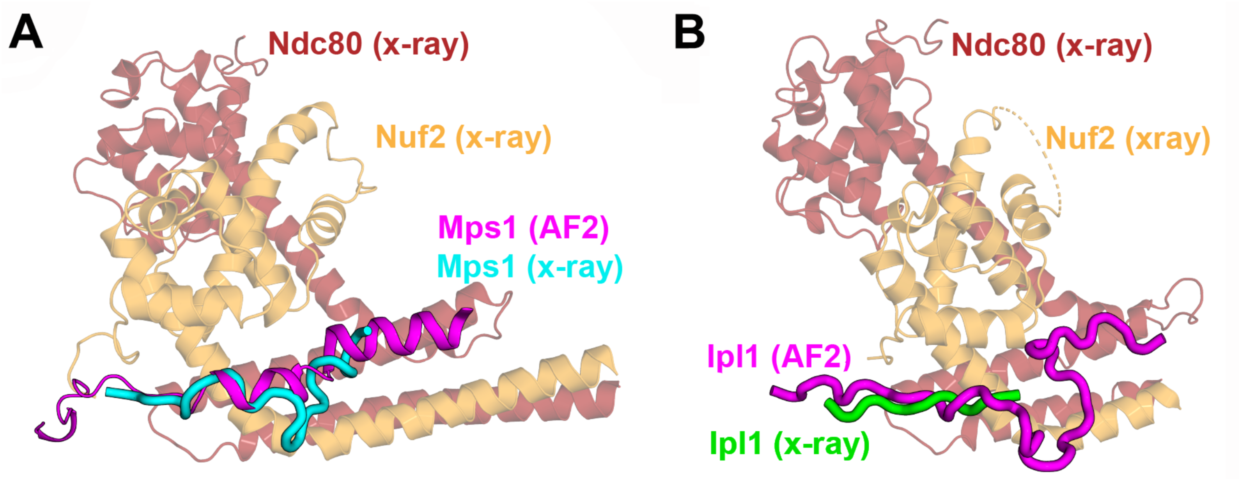

Figure S2. Comparison of the Ndc80c^{dwarf} - Mps1 and Ndc80c^{dwarf} - Ipl1 crystal structures with their respective AF2 predictions. Related to Figures 2, 4 and 5. (A) Superposition of the AF2 prediction of Mps1-bound head of Ndc80c^{dwarf} (Figure 2A) and the corresponding crystal structure (Figure 4). **(B)** Superposition of the AF2 prediction of Ipl1-bound head of Ndc80c^{dwarf} (Figure 2B) and the corresponding crystal structure (Figure 5).

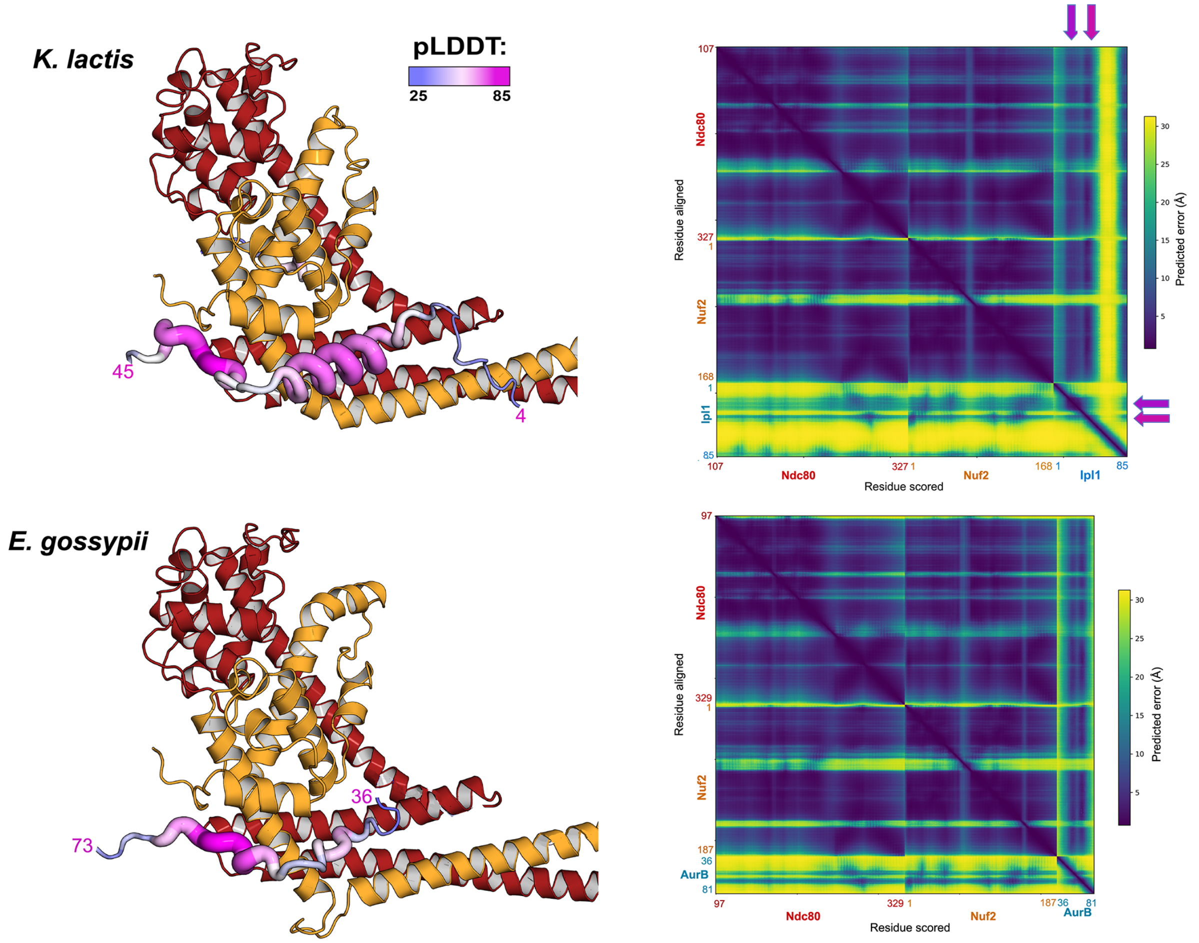

Figure S3. AF2 predictions for association of Ipl1/Aurora B with the head of Ndc80c, for *K. lactis* and *E. gossypii.* Related to Figures 2 and 5.
Shown are predictions for a relatively short segment from the N-terminal extension of the kinase, but we obtained the same predictions for longer segments. Details as in Figure 2. The Ipl1/Aurora B peptide sequences are 15-RHSLLQQSKLLSIRLSNAGKRP**SLSR**GHAHE-45 for *K. lactis* and 36-APPQARVQPGKGYRNPGKVLSPIRNQEMS**PGKR**PTLEISELKNVSP-81 for *E. gossypii*, where boldface shows the motif ending in the arginine that AF2 predicts will salt-bridge to a conserved aspartate in Ndc80. The *K. lactis* prediction was carried out with the full N-terminal extension (residues 1-85, as shown in the pAE plot), but only 15 to 45 are shown in the structure illustration, to avoid clutter from random chain. For *E. gossypii*, some of the lower-ranked predictions placed the LSPIR motif opposite the conserved aspartate. Both alternative might occur, contributing to avidity by vicinal rebinding; the SPIR motif is identical in its last four residues with the second motif in *S. cerevisiae* (see Figure 5).

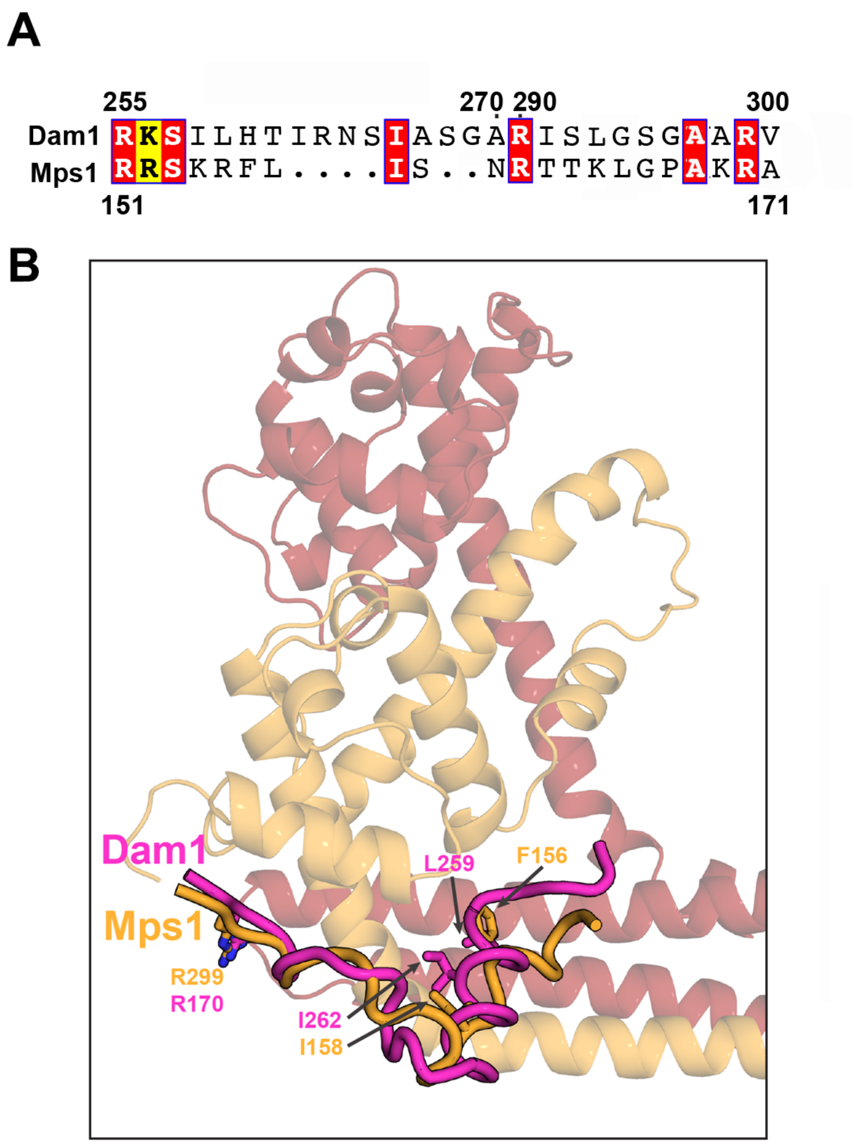

Figure S4. Comparison of Dam1 and Mps1 binding with Ndc80c. Related to Figure 4.
(A) Alignment of interacting sequences. Note that in designing the chimera for the crystal structure, we deleted the long loop between residues 270 and 290 in Dam1. (**B**) Cartoon representation of the head region of Ndc80c (Ndc80 in red, Nuf2 in yellow), with ribbon representation of the Dam1 and Mps1 peptides, from the respective crystal structures of the Ndc80^{dwarf} chimeras. Side chains of key hydrophobic residues from each chain and of the invariant arginine are in stick representation.

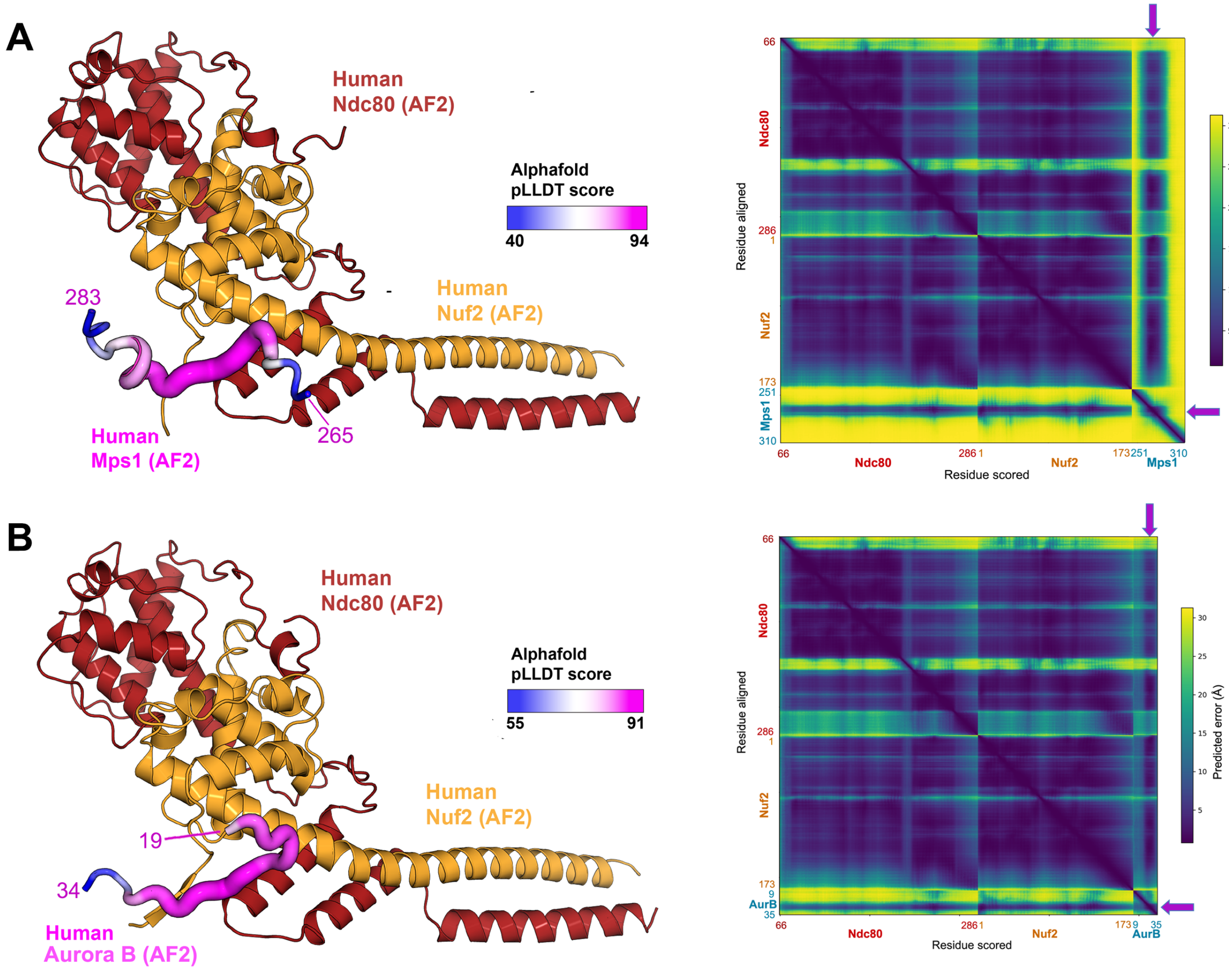

Figure S5. AF2 predictions for human Mps1 and Aurora B association with the head of human Ndc80c. Related to Figures 2, 4 and 5.

(A) AF2 multimer prediction for the Ndc80/Nuf2 CH domains and segment from human Mps1 "middle region" (265-TKQSC**PFGR**VPVNLLNSPDCD-285; tetrapeptide terminating in invariant arginine in boldface). The AF2 run and the pAE plot to the right included residues 251-310; only 265-285 are shown in the structure representation, to avoid clutter from disordered regions. Magenta arrows show residues corresponding to high pLLDT-score residues in the structure representation. **(B)** AF2 multimer prediction for the Ndc80/Nuf2 CH domains and segment from the N-terminal extension of human Aurora B (19-SGLST**LPQR**VLRKEPV-34: tetrapeptide terminating in invariant arginine in boldface). The pAE plot on the right included residues 9-35. AF2 predictions that included nearly the full N-terminal extension of human Aurora B gave the same result for these residues, with no confident prediction for the rest of the chain. Magenta arrows show residues corresponding to high pLLDT-score residues in the structure representation.

	Ndc80:Mps1-Nuf2	Ndc80:Ipl1-Nuf2
**Data collection**		
Space group	$C222_1$	$P4_32_12$
Cell dimensions		
a, b, c (Å)	72.65 168.30 230.214	114.76, 114.76, 423.84
α, β, γ (º)	90.0 90.0 90.0	90.0, 90.0, 90
STARANISO analysis	Diff. limit #1: 3.728, (1.0, 0.0, 0.0), **a***	Diff. limit #1: 6.237, (1.0, 0.0, 0.0), **a***
Diffraction limits (Å) [a]	Diff limit #2: 3.542, (0.0, 1.0, 0.0), **b***	Diff. limit #2: 6.237, (0.0, 1.0, 0.0), **b***
	Diff limit #3: 2.912, (0.0, 0.0, 1.0), **c***	Diff. limit #3: 3.361, (0.0, 0.0, 1.0), **c***
	Eigenvalue #1: 141.07, (1.0, 0.0, 0.0), **a***	Eigenvalue #1: 107.1, (1.0, 0.0, 0.0), **a***
Anisotropic tensor (Å^2) [b]	Eigenvalue#2: 141.37, (0.0, 1.0, 0.0), **b***	Eigenvalue #2: 107.1, (0.0, 1.0, 0.0), **b***
	Eigenvalue#3: 65.62, (0.0, 0.0, 1.0), **c***	Eigenvalue #3: 48.19, (0.0, 0.0, 1.0), **c***
Wavelength (Å)	1.03094	1.0309
Resolution (Å) [c]	115.1–3.03 (3.398-3.03)	48.53–3.95 (4.48–3.95)
R_{merge} (%)	15.5 (85.3)	55.6 (390.0)
R_{meas} (%)	18.1 (103.9)	57.3 (400.6)
R_{pim} (%)	0.68 (63.7)	13.5 (90.9)
I/σI	5.3 (1.6)	6.7 (1.6)
$CC_{1/2}$	0.99 (0.68)	0.99 (0.61)
Completeness spherical (%)	64.9 (11.2)	42.6 (10.5)
Completeness ellipsoidal(%)	88.9 (48.1)	89.0 (76.6)
Total number of observations	111103 (3752)	191124 (14902)
Unique reflections	18,187 (909)	10,971(554)
Redundancy	6.1 (4.1)	17.4 (19)
**Refinement**		
Resolution (Å)	115.1–3.21 (3.41–3.21)	48.53–3.95 (4.35–3.95)
No. of reflections used	16929 (735)	10971 (554)
Reflections used for R-free	890 (39)	559 (32)
R_{work} / R_{free} (%)	24.19 (27.32) / 35.19 (38.64)	26.67 / 32.66 (35.4 / 43.2)
**Model statistics**		
No. of non-hydrogen atoms	5725	11,265
B factors		
Average <B> (Å^2)	87.71	162.25
<B_i-B_j> (Å^2)	9.44	12
R.m.s deviations		
Bond lengths (Å)	0.001	0.0057
Bond angles (º)	0.35	1.13
Rotamer outliers (%)	0.15	0.08
Ramachandran angles		
Favored (%)	96.36	96.46
Outliers (%)	0	0.15
MolProbity clash score	4.63	16.5
PDB-ID	8V10	8V11

[a] Diffraction limits (Å) and corresponding principal axes of the ellipsoid fitted to the diffraction cut-off surface as direction cosines in the orthogonal basis (standard PDB convention), and in terms of reciprocal unit-cell vectors.
[b] Eigenvalues of overall anisotropy tensor on |F|s (Å^2) and corresponding eigenvectors of the overall anisotropy tensor as direction cosines in the orthogonal basis (standard PDB convention), and in terms of reciprocal unit-cell vectors.
[c] Highest resolution shell is shown in parenthesis.

Table S1. X-ray data collection and structure refinement statistics. Related to Figures 4 and 5 and STAR Methods.
